# Real-Time Learning and Monitoring System in Fighting against SARS-CoV-2 in a Private Indoor Environment

**DOI:** 10.3390/s22187001

**Published:** 2022-09-15

**Authors:** Serdar Erişen

**Affiliations:** Department of Architecture, Atılım University, Ankara 06830, Turkey; serdar.erisen@atilim.edu.tr

**Keywords:** SARS-CoV-2, real-time learning and monitoring, big data, indoor air, user activity, infection transmission control

## Abstract

The SARS-CoV-2 virus has posed formidable challenges that must be tackled through scientific and technological investigations on each environmental scale. This research aims to learn and report about the current state of user activities, in real-time, in a specially designed private indoor environment with sensors in infection transmission control of SARS-CoV-2. Thus, a real-time learning system that evolves and updates with each incoming piece of data from the environment is developed to predict user activities categorized for remote monitoring. Accordingly, various experiments are conducted in the private indoor space. Multiple sensors, with their inputs, are analyzed through the experiments. The experiment environment, installed with microgrids and Internet of Things (IoT) devices, has provided correlating data of various sensors from that special care context during the pandemic. The data is applied to classify user activities and develop a real-time learning and monitoring system to predict the IoT data. The microgrids were operated with the real-time learning system developed by comprehensive experiments on classification learning, regression learning, Error-Correcting Output Codes (ECOC), and deep learning models. With the help of machine learning experiments, data optimization, and the multilayered-tandem organization of the developed neural networks, the efficiency of this real-time monitoring system increases in learning the activity of users and predicting their actions, which are reported as feedback on the monitoring interfaces. The developed learning system predicts the real-time IoT data, accurately, in less than 5 milliseconds and generates big data that can be deployed for different usages in larger-scale facilities, networks, and e-health services.

## 1. Introduction

The COVID-19 disease, caused by the SARS-CoV-2 virus, was declared as a global pandemic in March 2020 [1]. The pandemic has forced several scientific and technological investigations to discover possible solutions [2,3,4], which also made this research inspect and apply technological advancements in the environment built for healthcare. The capacities of hospitals have been challenged by the SARS-CoV-2, and other healthcare services, such as surgery operations, have also been influenced negatively due to the decreasing number of allocated beds in public healthcare facilities [5]. This compelled us to investigate infection transmission control, at the private scale, by providing big data to be generated with the help of sensors, IoT devices, and learning models that can also be applied for e-health and smart health systems [6,7].

Motivated by the earlier research in constructing intelligent environments with smart systems operated with learning models, potential crowdsourcing and data processing methods, via sensors, are surveyed for possible usage scenarios [8] (p. 115) to generate big data when fighting against the SARS-CoV-2. The widely used application and smart healthcare system “Life Fits into Home” has also inspired this research to investigate the significance of monitoring for infection control at the private scale [9]. In that regard, the special care contexts are found as significant to be monitored for infection transmission control, inside private indoor spaces, by closer surveillance against COVID-19 [4,10,11].

This research has aimed to generate and process big data that belongs to various sensors, such as gas, temperature, humidity, and motion sensors, which are seen as critical for infection transmission control in private indoor spaces during the pandemic. Furthermore, the research attempts to deploy multiple sensors and analyzes their inputs accordingly to find correlating data about user activities and data from an indoor environment. Thus, the research has aimed to apply machine learning and deep learning models to better find correlating changes and primary classifications in these acquired data. The classified user activities, with inputs from the experiment environment, are used to develop a real-time learning and remote monitoring system to predict user activities that are seen as critical for infection transmission control and wellbeing in indoor environments.

Accordingly, the learning system is applied to report the current state and activities of the participants in the experiment environment. The research also surveys different usage scenarios applying sensors and learning models to develop an IoT-based real-time learning and monitoring system in infection transmission control. In the scope of the research, the selected experiment environment, designed for users who needed intense care at the private scale during the pandemic, was installed with microgrids, sensors, and Internet of Things (IoT) devices to produce real-time IoT data to be monitored instantly by the real-time learning and monitoring system, which is believed to decrease the interaction among users.

The indoor environment with sensors has enabled the generation, acquisition, and processing of lightweight and ubiquitous big data regarding the air quality, temperature, and humidity of the indoor space, together with user activity and rated health state values, to be registered to the e-health systems with less digital storage concerns. Stepwise experimental analyses are conducted via machine learning and deep learning models to recognize correlations between different sensor values, which are used to learn, classify, and predict user activities together with the environmental data. Thus, the learning system is also developed, with the help of machine learning models, to detect the correlating data of sensors. The system includes multilayered trained neural networks that are configured and operated, subsequently, to save the incoming/transferred data that the previous model cannot register to update the system. This unique configuration of the learning models increases the efficiency of saving and updating the system’s training and validation data. The training and validation data, as well as the neural networks in the system, evolve by each update without any need for further optimization. The outcomes of the research reveal that the system helps generate and process big data efficiently to alerting caregivers, in real-time, from the indoor environment, which is organized for palliative care at the private scale. The real-time learning system predicts each incoming real-time data as recorded by categorical user activities in 2–5 ms on average. The acquired and predicted data are provided, ubiquitously, to the IoT cloud that is publicly monitored by caregivers and doctors for infection transmission control in the fight against SARS-CoV-2.

Thus, the main contributions of the research to the literature can be briefly summarized: a unique real-time learning and monitoring system is developed for predicting user activities, and the system updates itself with each incoming real-time input. In developing the system, the spatial design and configuration of sensors in the indoor environment is one genuine aspect in generating and validating the correlational data about user activities. The spatial configuration of sensors, applied machine learning, deep learning models, error regularization, and data optimization algorithms help to validate the datasets and each incoming real-time input from the environment. Therefore, the developed learning system evolves with each update autonomously. The system is proven fast and efficient in predicting and reporting the current state of the patients and experimental actions that are classified and seen as critical to decrease the interaction among users for infection transmission control and wellbeing during COVID-19.

The remainder of this manuscript is organized as follows: Section 2 discusses the background research with related works and theories. The methods applied for this research with the material and models are presented in Section 3. Section 3 also provides the theoretical framework of the developed system with communication and connection details and calculations on the initial IoT data. Section 4 provides details about the results of conducted experiments. Section 5 discusses the outcomes of experiments in assessing the correlating IoT data, developing learning models, and the real-time learning system that is evaluated in the scope of the generation of big data for infection control and smart healthcare. Section 6 briefly concludes the research.

## 2. Background Research

The pandemic has challenged the capacities of healthcare facilities. Thus, further big data generation and processing have been needed with deep analyses of data from different cases and circumstances [5]. Significant surveys and research, including Healthcare IoT projects, classify various cases into personal and clinical categories in their physical environments and in dealing with the SARS-CoV-2 [4,5,12]. This distinction has raised the research question on the methods required for designing a variety of spatial usage scenarios, using the appropriate technologies and design considerations, for remote monitoring in the scope of smart health systems.

Microgrids and nanogrids are the primary building blocks of smart grids and the infrastructural development of smart systems [13,14,15]. Microgrids with sensors generate big data, and they are thought to be multiplied at the nodes of networks of e-health, smart healthcare, and monitoring systems [7,8,16,17,18]. Smart healthcare and smart city infrastructure can also be extended via the usage of microgrids and smart grids in the built environment [8,19]. Furthermore, the deployment of microgrids with sensors and IoT-based systems, at the residential scale, is thought to encourage the active participation of users [17,20] for smart healthcare monitoring and infection control against COVID-19 [12]. Thus, this research regards the significance of the active participation of users and patients at the residential scale with the help of microgrids [4,16,20,21]. This theory has extended to investigate different ways in which the usage of smart sensing systems and learning models can be developed, at the private scale, for special care [10,11,22,23].

The rise of smart systems, employed with sensors and artificial intelligence (AI) in buildings, has also played a key role in fulfilling the special needs of self-care and special care [10,23]. Moreover, IoT technologies and learning models have significant application potential in monitoring tasks and healthcare in smart spaces [4,11,21,24]. The IoT data from indoor spaces can also be crowdsourced by applying remote monitoring systems for smart healthcare services [7,16,24], and in most recent applications, the technologies are generally used for real-time monitoring [21,25,26].

The study has also surveyed the design constraints and parameters for the spatiotemporal configuration of automated systems in indoor environments, in generating real-time IoT data, to learn user activity. The geometrical configurations of systems in real-time data generation are also crucial in grasping geometric mapping-based techniques that can be modified into location-based setups of smart spaces [12,27,28]. Thus, the design and construction of intelligent architectural environments, with the physical configuration of smart systems and IoT technologies for smart health services, motivate this research further in the transformation of buildings with the accompanying rise in technology and AI for monitoring the user activity and environmental facts in real-time [8,29,30,31].

Accordingly, the usages of real-time learning models and smart systems, deployed with sensors, cameras, imaging devices, wearables, and personal devices for recognizing user movements, locations, and behavioral patterns in the physical constraints of buildings and indoor spaces, are also surveyed [21,22,23,32,33,34,35]. Thus, motion sensors are found to be crucial in keeping the critical distance between non-infected people and infected persons or caregivers in enclosed spaces. They also help to have crowdsourced data for e-health and smart health platforms about scattering, as well as the prevention of infection transmission during the COVID-19 pandemic [34,36]. The rising challenges of SARS-CoV-2 have also motivated investigations on the use of other sensors for monitoring the air quality in indoor spaces and for determining the activity pattern of people [37,38,39,40]. Moreover, real-time learning models have been applied in various research and medical tests in the detection of the SARS-CoV-2 virus [41,42,43,44,45].

The environmental parameters related to the air quality, temperature, humidity, and behavior prediction by learning movement patterns and user activity in indoor environments via AI models are also crucial for addressing the challenges of the pandemic and for providing special care conditions for the elderly and asthma patients [4,10,17,20,23,35,36,37,38,39,40,46,47,48]. Besides other sensor systems and wireless connections, wearables and Bluetooth devices are also used to monitor data from indoor environments [27,49,50]. Personal devices, as well as distance-based precautions by remote control or voice control, operated via language processing and Internet connections, have also gained importance in infection transmission control [23]. Furthermore, smart cards and smart personal data for public healthcare can be desirable for personal usage in dealing with the pandemic and having access to larger-scale smart health systems [16]. Thus, these technologies and models can also contribute to smart healthcare and logistics [12]. In addition to IoT and cloud computing, the abovementioned technologies and devices provide information to predict the usage patterns that are processed through machine learning (ML), deep learning, blockchain technologies, extreme learning machines (ELM), real-time learning system (RLS), and multi-task learning models [2,3,21,26,33,35,51,52,53,54,55,56,57].

The studies on healthcare monitoring and secure e-health systems emphasize the diverse set of IoT communications and applications, including healthcare by wearables, such as accelerometers, gyroscopes, smartphones, Global Positioning System (GPS) devices, motion sensors, and microphones [16,46,58,59,60]. Moreover, contact tracing applications implementing centralized and decentralized approaches have become significant in China, South Korea, Spain, Italy, and Singapore in dealing with symptomatic and asymptomatic COVID-19 cases [9,34]. To address the advanced inquiries on IoT-based smart healthcare systems and the role of smart grids in large-scale transformations and services, remote monitoring and the Internet of Healthcare Things can provide further possible solutions [55,58]. These approaches have great implications for the advancement of smart e-health systems, which can also be developed at the housing level for tackling the particular circumstances of diseases and healthcare.

Essentially, the increasing capabilities of microgrids with sensors and IoT devices, operated with real-time learning, monitoring, and prediction models, can generate big data to enable such large-scale infrastructural transformations through energy-efficient systems and real-time learning models [8,54]. Therefore, this research investigates how the physical setup of task-oriented microgrids can derive big data by formulating learning models and algorithms of different versions of “efficient online learning systems” [54]. These efforts are also considered as the groundwork, to be deployed in new indoor environments, for designing and constructing intelligent spaces and buildings providing big data for healthcare [6,11,12,34,49,58].

## 3. Materials and Methods

**Figure 1:**
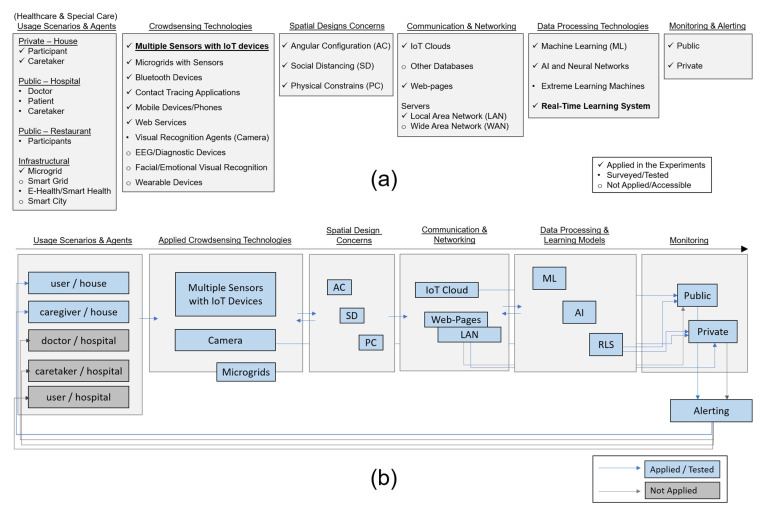
(**a**) Surveyed technologies and spatial usage scenarios for (**b**) the applied methodology and the scope of the research.

The distinction between the private and public spaces for healthcare is primarily considered for the methodology and scope of this research [12] (Figure 1). In that regard, the research first applies to investigate possible usage scenarios by taking into account the space considerations and participants for infection transmission control during the SARS-CoV-2 pandemic [4,5,8,9,12,19,38]. The surveyed technologies from the background research are also evaluated to have design and technological approaches for addressing different spatial usages and needs (Figure 1a). Regarding the problems caused by COVID-19 and the significance of infection control at the private scale [4,9], the study has aimed to deploy appropriate monitoring mechanisms for infection transmission control in indoor environments [40] to decrease the interactions among users during the pandemic. Thus, the objective of the research can be briefly declared to crowdsource multiple data points from different sensors in a private indoor environment for special care and to learn and predict the categorized data about user activities by developing an efficient real-time learning system for tracing the correlating changes in these data. In the scope of the research, the processed data are also seen as experimentally novel to follow the persisting circumstances that are recognized as critical to alert caregivers during the pandemic (Figure 1b).

Accordingly, the research project was initiated with the selection of participants and appropriate technologies in the designation of an environment at the private scale (Figure 1). The selected room of one apartment unit was designed as an indoor environment for palliative care and monitoring infection transmission control during the pandemic. Participants are the inhabitants of the house, who are a married couple over 65 years of age, and one is an asthma patient.

Among the surveyed technologies and design considerations to develop monitoring technologies, microgrids, as the primary keystones of smart grids and services [13,14,15] are found to be appropriate for private scale usage, having greater potential to be integrated into the smart infrastructure of larger-scale networking and smart health applications. Therefore, the study decided to operate microgrids, with sensors connected to an IoT cloud platform [61], to generate and process big real-time data for training and developing learning models, as well as by considering the physical configuration parameters of the experiment environment (Figure 1). The microgrids acquire crowdsourced data by sensors that are also measuring the gas levels and air index values regarding the critical condition of the asthma patient [47,48].

As one of the primary methods of this research, the correlations among data of multiple sensors are followed, and these correlations are analyzed to determine categorized activities. These categorical activities are also seen as experimentally valuable, to track the user occupation by following the environmental data and considering the gas sensor and air index values, in correlation with the user movement with regard to the spatial configuration of sensors. The acquired inputs are correlated and classified with the help of the spatial configuration pattern, as well as machine learning and deep learning models, in developing the real-time learning system. In short, with the special spatial design of sensors, as well as the developed machine learning and deep learning models, the research is designated to generate and validate the initial experimental datasets and exclude errors by error-correcting algorithms that are used to develop an autonomous real-time learning and monitoring system. The system operates and evolves by applying data validation and optimization steps.

In the spatial configuration of smart systems, projects for the elderly and patients with Alzheimer’s and dementia have also inspired this research to consider the purpose of monitoring and learning about user occupancy [17,62] in infection transmission control. This was achieved by the method of motion tracking and tracing the correlated activity pattern of the participants in the indoor space with specially designed microgrids with multiple sensors and IoT devices. Thus, distance-based motion tracking is applied as a method of following user activities in the experiment room, wherein the two designed microgrids with sensors were configured with different angles and synchronized with the IoT cloud [61]. Spatial design constraints are studied through the angular configuration of the applied technologies to find correlating motion tracking data from multiple sensors. Social distancing is primarily considered with the physical constraints and furniture layout of the selected experiment environment to decrease risky interactions for infection transmission control during the pandemic.

For networked communication, IoT devices send and acquire real-time IoT data and deliver inputs for real-time monitoring. The installed smart systems provide IoT data for remote monitoring activity of users for infection transmission control and wellbeing during the pandemic. Thus, the IoT devices were connected to the IoT cloud that caregivers and doctors could view instantly during the pandemic, as it can also be viewed publicly. Additionally, a Local-Area-Network (LAN) connection is provided so that it can be accessed by caretakers and hospitals in the closer territory.

The acquired IoT data are applied to advance the real-time learning system. Among different technological applications, affordable, fast, and efficient methods and models are explored to develop the real-time learning system. The developed system is deployed to monitor the acquired data that facilitates remote monitoring via the IoT cloud, predicts the current state of user activities, and provides feedback about the activity to the IoT cloud. The acquired predictions are processed to send private electronic mail alerts about critical circumstances. The experiments were conducted for one year during the SARS-CoV-2 pandemic and ended in November 2021.

The applied methodology has also aimed to re-evaluate the outcomes of learning models in the scope of the surveyed spatial usages and technologies in the discussion section with the advantages and limitations (Figure 1b). Therefore, the study also describes the application of other alternative usage scenarios regarding the applied methods, technologies, models, and types of monitoring in the scope of spaces and services for e-health and smart health systems and networks.

### 3.1. Features of the Microgrids: Microcontrollers with Sensors and IoT Devices

To briefly mention them, three different microcontrollers with sensors and IoT devices are deployed to acquire multiple data from different sensors that are to be analyzed for any correlation with each other via user action. Two of the microgrids were installed with IoT technologies within the special physical configuration of the experiment room. All three microgrids and ESP32 devices (Espressif Systems, Shanghai, China) are hard-coded using Arduino IDE software (Italy). The connections of sensors in each microcontroller are distributed by considering the workloads with microgrids, as presented in Table 1, along with other technologies tested during the experiments.

According to this, “Microgrid X” is built on one microcontroller, including an ultrasonic sensor to track motion. The challenges posed by the SARS-CoV-2 pandemic have made the use of gas sensors vital [37,38,39,40,47,48]. One remote controller was applied as a distance-based precaution to get user-defined health-state values ubiquitously. Accordingly, the rating ranges are set from 1 to 5 with the same controller buttons; otherwise, the microgrid sends the default value (Table 2).

“Microgrid Y” is also built on one microcontroller with one ultrasonic sensor to measure the distance from moving agents. Thus, Microgrid X and Microgrid Y were chosen to be installed in the physical configuration of the experiment environment to have the correlating motion-tracking data of user activities. A temperature and humidity sensor is applied to provide ubiquitous data about the indoor space beside other display and internet connection capabilities.

“Microgrid Z” is built on another identical microcontroller, designed as a discrete model that is free from physical configuration constraints. It works with Bluetooth, which is controlled by smartphone applications using numeric, text, and sound commands, and operates wireless devices by enabling multi-port uses. The connected Bluetooth device recognizes other paired devices and mobile technologies, which are also deployed to trace critical contacts by getting commands from other mobile devices [9].

### 3.2. Spatial Design Concerns of the Experiment Room

**Figure 2:**
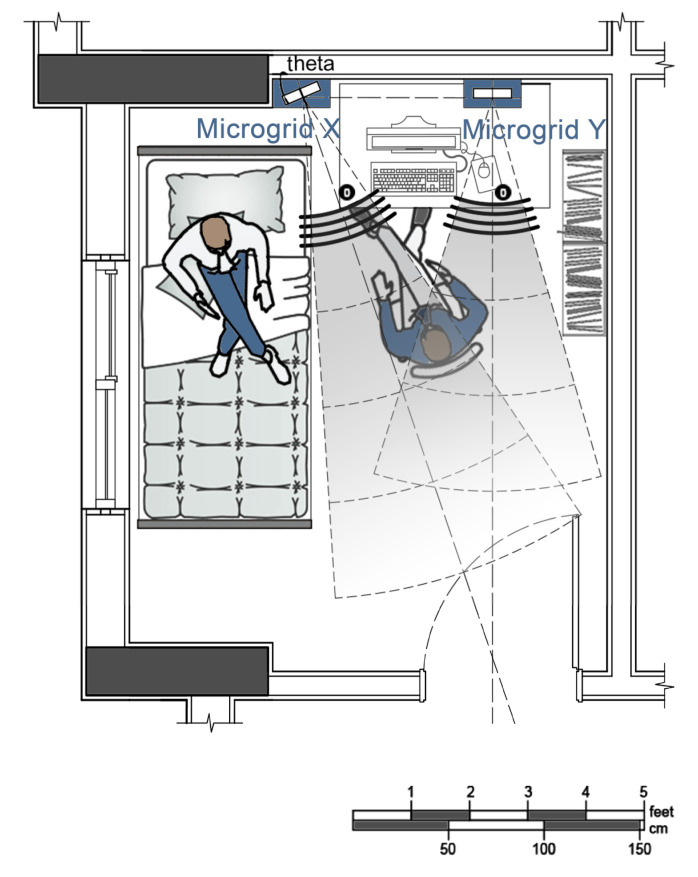
Microgrids installed in the experiment room with a spatial configuration.

The geometrical configurations of Microgrid X and Microgrid Y are designed to obtain certain location information and data about user activity and validate each motion tracking input with regard to the other. Thus, the microgrids were installed in an angular configuration to correlate the measured distances. Accordingly, the physical setup is designed to provide a location pattern such that Microgrid X, tilted with an angular configuration, *theta* (*θ*), checks the dimension, tracked by Microgrid Y, with regard to the triangulation of the measured distances (Figure 2). In other words, the spatial configuration of microgrids with sensors has not only allowed for tracing the correlating data of multiple sensors but also provides the basis to validate and predict the motion tracking data of each microgrid with regard to the other.

In this configuration pattern, the entrance of the room and the location of the bed of the patient were considered primarily to find out critical measurements during infection transmission control. Therefore, Microgrid Y was set on an axis that had a direct view of the entrance of the experiment room, and Microgrid X was oriented toward the diagonal axis of the room, with the longest viewing distance from the entrance (Figure 2). The measurement origins for Microgrid X and Microgrid Y were then fixed based on the placement of other furniture used by the patient, and the layout of other furniture was configured according to these constraints. Moreover, the measures for distance from the patient and the furniture that patient uses were decided as 150 cm for infection transmission and interaction control. These configuration parametrizations were applied to track a patient in need of care in the experiment room and to recognize a person who was visiting, taking care of the patient, or operating some equipment in the room (Figure 2).

The physical setup of the experiment room aims to obtain critical behavior patterns from the immediate environment around the patient by using microgrids to generate real-time IoT data. The outcomes are monitored by the learning system, which is to be applied to smart health systems.

### 3.3. Communication Organization of the Microgrids for the IoT Data

**Figure 3:**
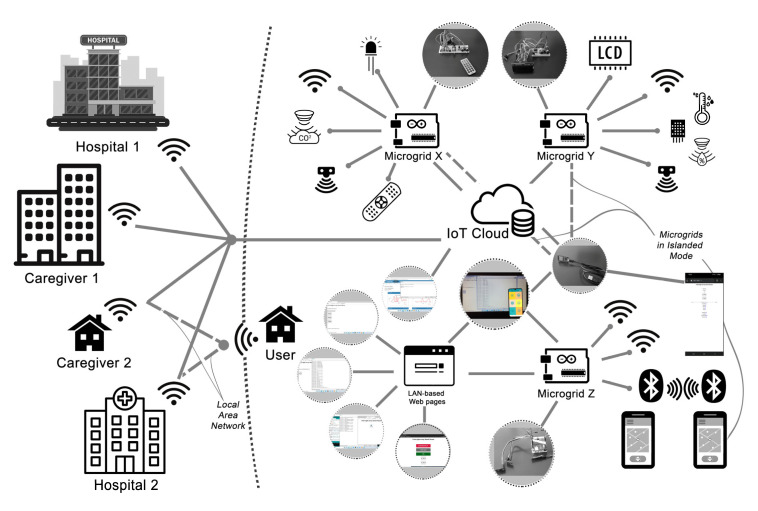
Nodes of sensors and communication schemata of the microgrids.

Connections to the IoT cloud [61] and other IoT devices have been provided through the connected wireless devices of the microgrids via the wireless network connection with IEEE 802.11 standards. The correlating data from Microgrid X and Y have been sent to six different IoT channel feeds. The serial data have also been acquired and merged by the IoT device, ESP32, for better communication (Figure 3). The developed real-time learning system has also been operated to merge and synchronize the acquired real-time data, which have, again, been sent to the same IoT channel via the same protocol in the ongoing experiments. The ESP32 device has also been hard-coded and deployed further for the local communication between the microgrids with IoT devices in the islanded mode without an Internet connection.

Microgrid Z is connected to its web server and provides a distinct rating and command system, similar to Microgrid X, to collect comparable user data. Based on the same LAN connection of its web-server, which is designed in Hyper-Text Markup Language (HTML) for various configurations of its web pages as a search on the capacities of Wi-Fi devices, this microgrid can be connected from other devices as an access point (AP) (Figure 3). Microgrid Z is designed as a portable device that can also sense other defined Bluetooth devices in its near territory. Thus, Microgrid Z is designated to be accessed from closer wireless areas that include territories of caregivers and a local hospital, and this connection is secured with a private network security key and Service Set Identifier (SSID) (Figure 3).

### 3.4. Applying the Learning Models on the IoT Data

The aim of applying machine learning models is to classify initial findings in the data of sensors that are observed to find critical correlations among motion, gas, temperature, and humidity sensors besides optimizing the crowdsourced data about user occupancy and interactions in the room. The acquired real-time IoT data from Microgrid X and Y are processed by learning models to develop a real-time learning and monitoring system for predicting the ongoing activity. Machine learning models are initially explored via classification and regression tasks, followed by Support Vector Machine (SVM) and ECOC models. Then, the same IoT data are processed via different deep learning neural networks, including Convolutional Neural Networks (CNN), Long-short Term Memory (LSTM), and Binary Layered Long-Short Term Memory (Bi-LSTM) neural networks, with and without batch normalization (Btc.N.). The initial experiments on the classification and regression models are related to user activity patterns and the physical configuration variables. In optimizing the trained neural networks, outcomes of experiments on machine learning models are also applied in the ceiling analyses of the deep learning models using error-correcting and data optimization algorithms.

### 3.5. The Real-Time Learning and Monitoring System

Finally, the real-time learning system is developed with an aim to report the current state and activities of the participants that are to be classified, according to the correlating changes, between sensors for infection transmission control in the experiment room. The system learns and predicts these classified activities to be alert for critical circumstances. Additionally, the remote monitoring system aims to minimize the interaction among users.

The optimized neural networks are operated, subsequently, for the real-time updating system that acquires and saves online data with high accuracy for a continuously updated dataset. Each learning model predicts the user activity and environmental factors that the previous models could not achieve and returns the labeled results to the IoT cloud that the caregivers can monitor. Thus, in the general architecture of the system, different types of deep learning networks are configured to respond, as flexibly as possible, to unknown online data.

The real-time data are instantly predicted and then introduced as newly updated data, either to the existing training or validation data, by the data optimization algorithm run in the system or saved for further analyses. The neural networks are also updated periodically with these new training and validation datasets, considering the increasing number of data updates. The system, again, controls the number of data updates to collect further highly precise online test data and re-train deep learning layers. This research has also enabled the assessment of the efficiency of the architecture of this evolving real-time learning system with different weighted prediction scores of the neural networks.

## 4. Experiments and Results

Following the methodology and methods, experiments were performed to generate and process correlational data of different sensors, which were sent as unique data to the IoT cloud [61] and used to develop learning models that run through a real-time learning and monitoring system efficiently (Figure 1b). Microgrids with various sensors were operated to create the initial dataset that is also processed to have the training, validation, and test datasets. The real-time IoT data from sensors are assessed to find strong correlations, in motion-tracking data and related data, about the air quality and other sensor states in the indoor space to identify the usage patterns. The initial IoT data are classified and processed through advanced machine learning techniques to optimize deep learning models and the training data. The developed deep learning models are organized within the real-time learning and monitoring system.

### 4.1. Initial Calculations on the IoT Data from Microgrids

**Figure 4:**
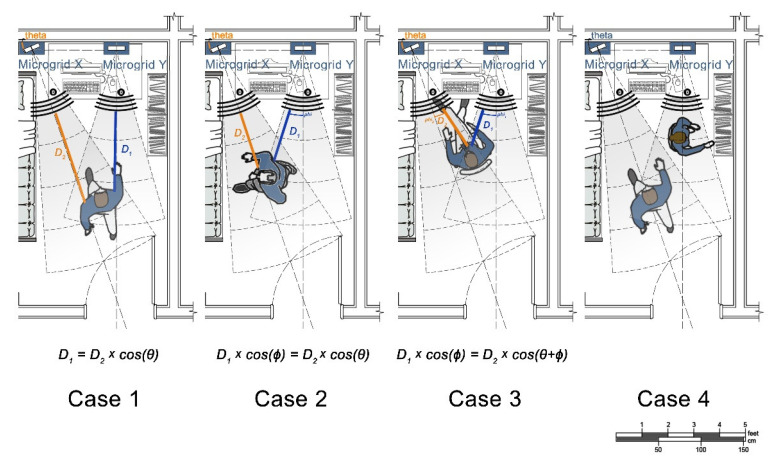
Evaluation of cases by spatial configuration and measurements.

The acquired IoT data from the cloud are, first, classified through motion tracking analyses. As illustrated in Figure 4, the four different cases are distinguished and recorded through specific measurements, from Microgrids X and Y, regarding the angle of physical configurations and the angles of agents with regard to the microgrids.

**Figure 5:**
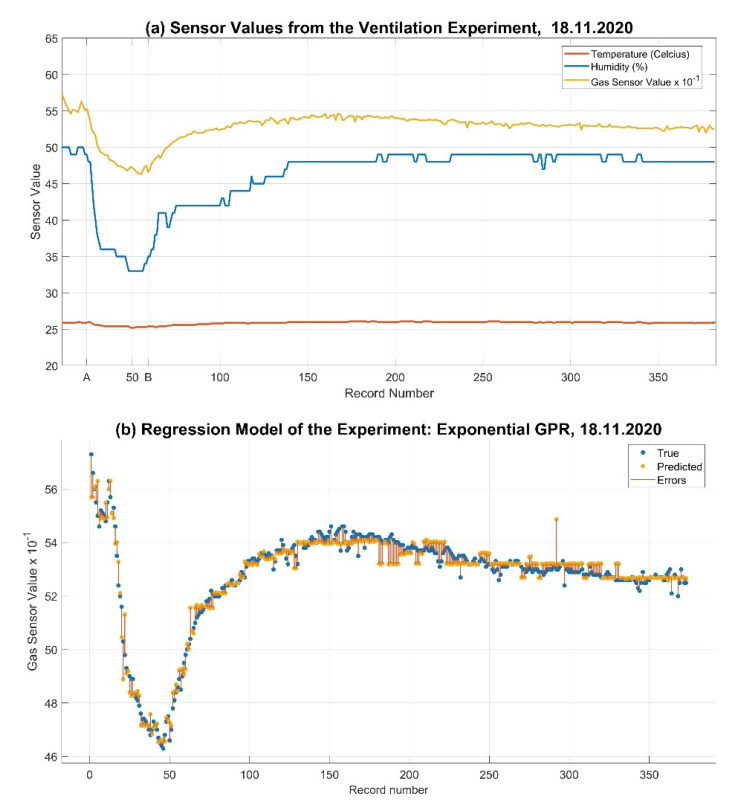
(**a**) Sensor values from the ventilation experiment and (**b**) its Regression Model.

In addition to the user activity and health–check values, correlating inputs regarding temperature, humidity, and gas sensor values are also investigated. The changes in temperature, humidity, and gas sensor values of the interior space were observed under different circumstances, as in the exemplar experiment, by providing natural ventilation to the well-heated room for 15 min on 18 November 2020, as illustrated in Figure 5a, with an outside temperature of 13.9 °C. In this experiment, the received temperature, humidity, and gas sensor values are found to be strongly correlated. Accordingly, machine learning and deep learning models are further applied for analyzing the correlating changes in indoor air, temperature, and humidity conditions besides movement behavior. Thus, a regression learning task is conducted, initially, to determine the best computation method for precisely calculating the changes of gas sensor values by natural ventilation of the indoor space (Table 3). MATLAB Regression Learner is run to predict the gas sensor values in correlation with temperature and humidity values, allocated as predictors, using 5-fold cross-validation. In this experiment, Gaussian Process Regression (GPR) returns the best Root-Mean-Squared-Error (RMSE) result, as illustrated in Figure 5b and Table 3.

### 4.2. Experiments on Machine Learning Models

Machine learning models are also applied to find and classify the correlating changes among different sensor values in the motion-tracking data. With the help of the physical configuration of the microgrids, the incoming inputs from different sensors are correlated and processed to also be classified by machine learning models. Thus, classification learning, regression learning, and ECOC Classification models are deployed to identify and predict the correlating data in classifying user activities.

#### 4.2.1. Classification Learning

**Figure 6:**
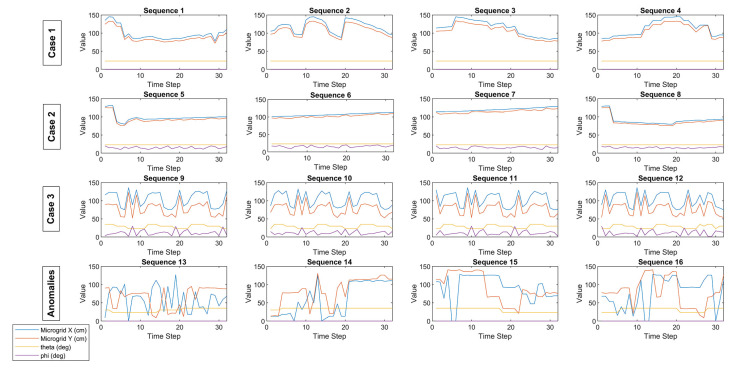
Initial Classification of the Movement Behavior of Users.

The given equations and configuration angles, between the microgrids in Figure 4, provide a basis to distinguish the three cases and regress the outcomes as correlational facts. However, some anomalies that do not fit any of the three equations in Figure 4 are classified as the fourth case (Figure 6). Thus, machine learning models are further applied to determine and validate these four classes, including minor differences and anomalies that cannot be classified by simpler equations (Figure 6). Based on the initial classifications, “MATLAB Classification Learner” is used to check the validity of each classification among Cases 1, 2, 3, and 4 (labeled as 0).

Therefore, in classification tasks, 1012-by-2 and 1012-by-4 inputs are introduced with their classification labels using 5-fold cross-validation. For optimizing the classification models, each model is experimented with using Principal Component Analysis (PCA) with an enabled variance of 0.95. Two different experiments are performed: In the first one, 1012-by-4 inputs include the configuration angles of Microgrids X and Y; in the second one, 1012-by-2 inputs are classified without the angle information (Table 4). The classification results are presented in Table 4.

**Figure 7:**
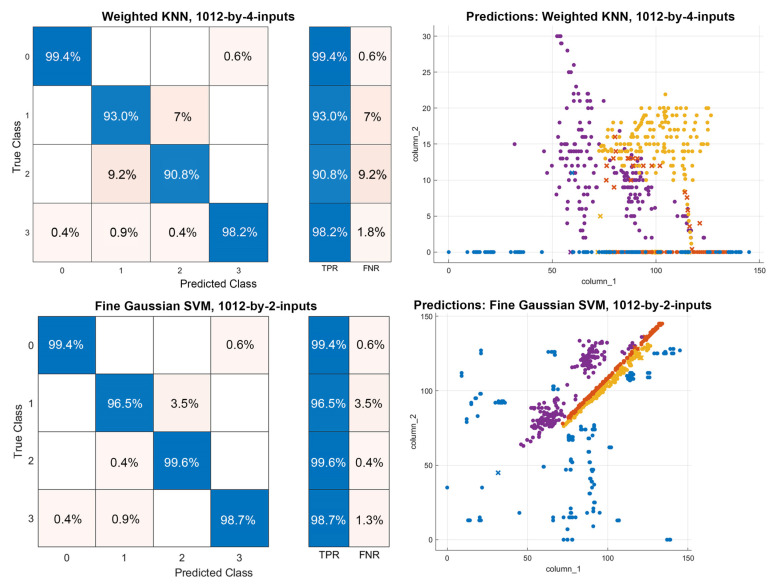
Exemplar confusion matrices and prediction plots for classification tasks.

Accordingly, the weighted K-means the Clustering for the Nearest Neighbor (KNN) algorithm returns the best outcome, with an average accuracy of 95.8, for two variables over the dataset with 1012-by-4 inputs (Figure 7). The fine Gaussian SVM method yields the best result with an average accuracy of 98.6 for the dataset with 1012-by-2 inputs (Figure 7).

#### 4.2.2. Regression Learning

Depending on the spatial configuration of microgrids, regression models are further deployed to validate and predict the motion tracking data of each microgrid. As regression models were also applied to follow the correlating changes between the air index and gas sensor values, further experiments are conducted to determine the precision in correlating changes in the user movements. Therefore, it is also aimed to provide outcomes about which learning model performs best in predicting the cases and correlating data with regard to the other.

Thus, the classified cases are further trained by using “MATLAB Regression Learner” to improve fast and efficient regression. Regression models are explored on the dataset with 2024 inputs, initially processed by MATLAB Classification Learner, to predict cases by considering the responses from Microgrid X and Y. Regression models are also explored to decide the priority among the microgrids through the correlating data that are compared with the RMSE results in Table 5 for optimizing the learning models. The data from each microgrid was tested in predicting the action by allocating the other as the predictor and using 5-fold cross-validation, with the following outcomes, in Table 5.

Thus, the regression learning models also return distinguished results in determining the anomalies differing from other cases and finding some classification errors.

#### 4.2.3. ECOC Classification Models with Different Learner Types

**Figure 8:**
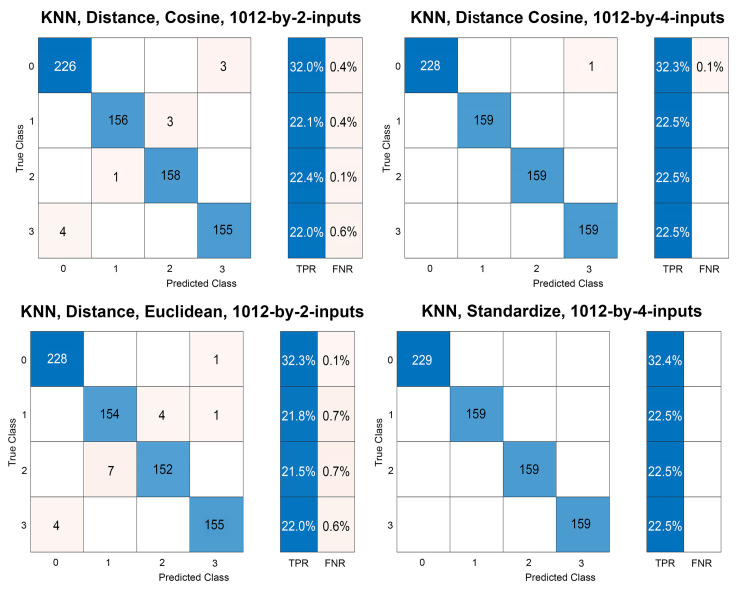
Confusion charts of ECOC models.

ECOC classification models are further applied for the experiments to have precise learning models and datasets that are validated through the correlations between the measured distances and critical angles, *theta* (*θ*) and *phi* (*Φ*). ECOC is one advanced machine learning method of SVM, for multiclass label classification, for more than two/binary classes. Thus, ECOC classification models are further applied to classify multiclass categories, including the information of configuration angles in the dataset. The previous datasets, including 1012-by-2 and 1012-by-4 inputs, are discretely introduced with and without cross-validation (Table 6). For obtaining the validation accuracy, MATLAB *fitcecoc* function allocates the training and validation data from the dataset [63]. In experimental cross-validation, the datasets are divided into randomly selected 706 (70%) inputs for training/cross-validation and 306 (30%) inputs for test datasets. Some results of the tested models are reported, in detail, in Table 6 and Figure 8.

### 4.3. Preparation of the Dataset with Classified User Activities

From the earlier experiments on machine learning models, 2024 (1012-by-2) and 4048 (1012-by-4, with angle information) inputs, with their classification labels, are classified and regressed. Further regression and classification tasks are applied, as in KNN and GPR algorithms, through SVM and ECOC classification to validate the correlating inputs among microgrids in the experimental datasets as discussed. Depending on the earlier classification experiments on the movement behavior with distinguished cases, as well as the correlations between gas sensor values, temperature and humidity values, and the inputs from the remote control, the acquired data are classified into ten different labeled activities (Table 7). These activities are seen as experimental, and persisting conditions are evaluated as critical, to be predicted and reported by the learning system, by following the correlation between multiple data from different sensors (Table 7).

For instance, while a person, as a caretaker, sits behind the patient/user, it is also observed that gas sensor values are high. In labeling the ventilation activity, moving for opening the windows and sequential changes in the air index and gas sensor values are observed as being correlated. Furthermore, higher and lower levels of temperature and humidity are labeled as critical for the comfort and wellbeing of users. The occupation of the room by its user is tracked by motion recognition and the active usage of remote control in the room (Table 7). Classified anomalies by the machine learning tasks are also used to validate and classify the critical activities, such as more than one person occupying the room, as in the classified sequences from 13 to 16 in Figure 6.

Similarly, moving towards the bed/patient and interacting around the bed of the patient are also labeled as critical activities regarding the criteria of social distancing for infection transmission control by following the correlation in the sequential changes of motion tracking data, as well as gas sensor values and rated user values. The earlier classified sequences by machine learning models are also used to classify these actions by finding the anomalies. More significantly, levels of gas sensor data and user-rated values are labeled as directly correlated, and they are seen as extremely crucial to be followed during the pandemic (Table 7). In the generation of the initial dataset, each categorized activity was also recorded by taking pictures to identify and validate the exact behavior besides getting the help of spatial configuration, especially in validating the motion tracking data.

Thus, 1872 (312-by-6) inputs with six different input types, including motion tracking, air quality, temperature, humidity, and crowdsourced health check values, recorded with ten differing user activity cases, were deployed to train deep learning models (Table 7). Accordingly, CNN, LSTM, and Bi-LSTM networks were further applied to learn the classified user activities (Table 7). After optimizing the learning models for the real-time learning and monitoring system, ongoing experiments were maintained to update the training data and learning models. The acquired data is applied to develop the real-time learning and monitoring system to predict user activities and update the training data of the learning models in the system. In the ongoing experiments, after having precise learning models to classify anomalies and the distinguished cases, the incoming inputs are validated with regard to the spatial configuration parameters of the microgrids. All computational experiments are performed in MATLAB in developing the learning models and the real-time learning system.

### 4.4. Experiments on Deep Neural Networks

Different deep neural networks, distinguished by their data types, are also experimented on with the same datasets to efficiently classify and learn the correlating sensor values and user activities. For instance, the datasets are processed to train CNN learning models as image data. Similarly, the datasets are also formatted as sequential data to observe the subsequent changes in the incoming inputs by sequence-based neural networks. Thus, sequential data are applied to train LSTM and Bi-LSTM neural networks to observe the difference between various neural networks that process different data types, which are explored in creating an efficient real-time learning system.

#### 4.4.1. Pretraining Deep Neural Networks

Initial datasets with 1012-by-2, 1012-by-4, and 312-by-6 inputs are prepared, discretely, as 1-by-2-by-1, 1-by-4-by-1, or 1-by-6-by-1 image data for CNN layers. The same datasets are prepared in cellular arrays of either 2-, 4-, or 6-by-n sequential data to be trained for sequence layers such as LSTM and Bi-LSTM neural networks. The datasets are divided into the sectors of 50%, 30%, and 20% of all data and arranged as training, validation, and test datasets, respectively. All neural networks were generated without any pretraining experiment at first, and the performance results of these neural networks are presented in Table 8 without any error regularization and correction. The neural networks were optimized, later, with an error regularization algorithm, which is applied for the ceiling analyses, and their training results are presented in Table 8.

#### 4.4.2. Error Regularization and Data Optimization

The outcomes of machine learning and deep learning models with minor errors are evaluated to develop an error regularization algorithm to swap the erroneous training and validation data of the earlier classifications in the datasets with new incoming inputs, in real-time, that are predicted with higher scores (Algorithm 1). Thus, false negatives and errors with the lowest prediction scores are excluded from the dataset by getting help from earlier machine learning tasks (Figure 7 and Figure 8). Predictions with lower scores, confused with other labels, are also indexed with their prediction scores; they are gated through functions of score thresholds to be swapped with training or validation data that have considerably higher scores (Algorithm 1). While excluding the erroneous data from the datasets that are swapped with new ones, the predicted classification results and true categorical classes are compared to prevent the replacement with any other unfitting prediction label (Algorithm 1). A similar algorithm is also applied for optimizing the existing data of the system by excluding the inputs with lower prediction scores.
**Algorithm 1** Pseudo-code for error regularization and data optimization[*YPred, score*] *= classify*(*DeepNNx*, *ValidationX*) [*Index, val] = find*(*YPred ~= ValidationY*) *for n =* [*index*] *   if max*(*score*(*n*) *< 0.5*)    * ValidationX*(*n*) *= []*;    * ValidationY*(*n*) *= []*;      *elseif max*(*score*(*n*) *> 0.5*)    * ValXSwap*(*n*) *= ValidationX(n);*      *for val*(*n*)*~=ValidationY*(*n*)         *find*(*TrainingX*(*n*)==*ValidationY*(*n*))      *end*    *TraXSwap*(*n*) *= TrainingX*(*n*);    * ValidationX*(*n*) *= TraXSwap*(*n*);    * TrainingX*(*n*) *= ValXSwap*(*n*);    *else*
      *…*, *fitcknn*(*ValidationX*(*n*), *ValidationY*(*n*), *…*), *…*    *end* *end*

### 4.5. Experimental Analyses and Outcomes of the Real-Time Learning and Monitoring System

**Figure 9:**
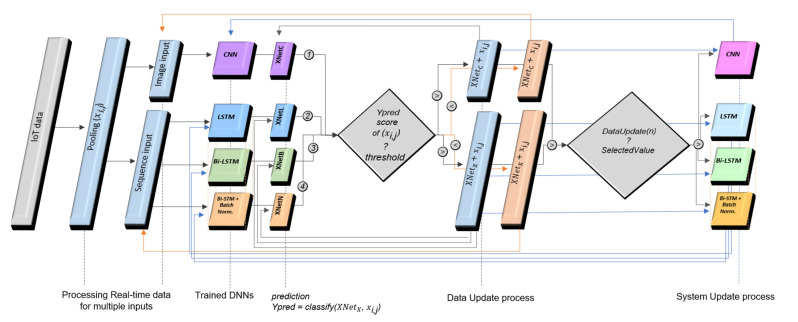
Model structure of real-time updating learning system, with deep neural networks ordered in the multilayered-tandem configuration.

The ceiling analyses of the neural networks are achieved by running the data optimization algorithm within each training session and repeating it until reaching optimal results in Table 8. After the ceiling analyses, the optimized neural networks are configured in the real-time updating learning system, based on their data types (Figure 9) and their performance results, regarding the duration of training sessions, practicality, and precision in prediction (Table 8). Then, the trained sequential neural networks are sequentially ordered in a multilayered-tandem configuration with their defined option and layers to increase the real-time efficiency in saving the IoT data. Overall, the updating neural network system is designed to classify and predict real-time data and exert the ones with higher prediction precisions into the training and validation datasets, as in the similar operations in the error regularization algorithm, though by setting higher prediction score thresholds (Figure 9).

For each new input, real-time data prediction for image and sequential learning layers are processed to have data updates (Table 9). For this processing, the predicted data is gated through conditional operations (Figure 9), and the threshold values are defined to evaluate each prediction score. Thus, thresholds indicate the prediction performance of the trained neural networks for each label in the ten categorized activities (Table 9). Accordingly, threshold values higher than 0.1, such as 0.89 or 0.8, are found appropriate in predicting the ten labeled categories precisely.

**Figure 10:**
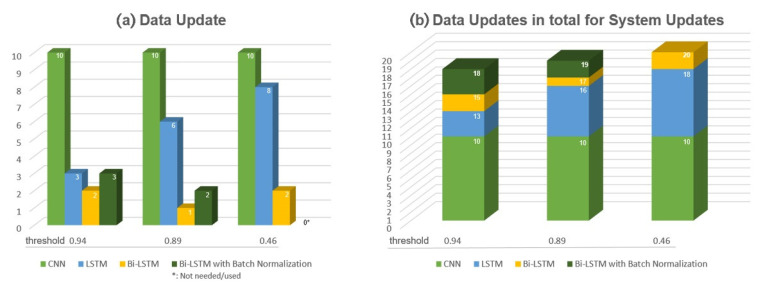
(**a**) Data Update Number in developing the Real-time Learning System (**b**) Data Updates in total for System Updates. *: Not needed/used.

The initial prediction performance of the system is also evaluated by setting the deciding threshold at different values in filtering the prediction scores of learning models, which are summarized in Figure 10. After conditional operations, the real-time data are either registered for updating the training and validation data, exchanged with the existing ones, or saved as distinct data when pooling for further analyses (Figure 9). The multilayered-tandem configuration in this real-time learning system is applied to save the transferred incoming real-time IoT inputs, which the previous layer could not predict. Thus, the special organization of neural networks in the system increases the number of data updates by backing up the first system layer to determine an update (Figure 9 and Figure 10 and Table 9). A certain amount of change in the value of data updates is balanced by applying the data optimization algorithm to remove the data with lower prediction scores and to re-train the developed learning models with these evolving training and validation datasets.

As illustrated in Figure 10 and Table 9, the efficiency of the proposed system increases with multilayered neural networks by backing up the previous deep learning models. Further analyses reveal the most significant advantage of the multilayered-tandem configuration of the neural networks: With data and system updates, the system maintains its accuracy without the need for further error regularization (Table 9). Particular experiments were conducted with users quarantined for infection transmission control, for five consecutive days, from 8–12 June 2021. The experiments reveal that the learning system achieves 99.97% prediction accuracy, on average, for a total of 6080 unknown real-time data with the assistance of the updated and evolved multilayered-tandem configuration of neural networks (Table 9). On 11 June 2021, for instance, a fully-developed CNN was operated by the system with a threshold value of 0.80, and the outcomes reveal that all the 1440 inputs were accurately predicted (Table 9).

**Figure 11:**
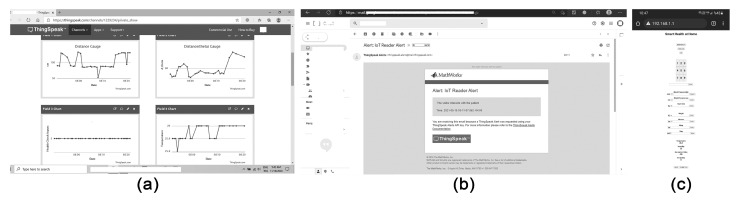
(**a**) The IoT cloud for public monitoring, (**b**) A private alert sent as an electronic mail, (**c**) LAN server of the system.

It can be concluded from the results that the rising efficiency of the system enables to provide instant predictions for each new activity as the system achieved 1391 correct predictions out of 1392 new inputs on 9 June 2021; 1342 accurate predictions out of 1343 new inputs on 10 June 2021 returned as feedback to the IoT cloud in monitoring the user activity (Table 9). The predicted labels of the recent user activity and their prediction scores in percentages appear on the IoT cloud platform, which can be publicly monitored (Figure 11a). It is crucial to have instant feedback about perpetually predicted user activities and precision values as a lifecycle pattern for caregivers with regard to the user occupation and environmental facts. Following the predicted activities during the experiments, it can also be concluded that, with the help of the remote monitoring system, the need for physical observation was reduced except for necessary interactions (Table 9).

Acquired inputs, when predicted correctly, are further processed to update the training and validation datasets, which also increase the system’s efficiency. The increased efficiency of the multilayered learning and monitoring system in predicting user activities has allowed the recognition of recurring prediction labels, which are evaluated to send instant private alerts. For instance, two people interacting for more than 120 s or with health state values below three throughout a day, three hours-long high or low temperatures, and 30 min-long high gas sensor values are recognized as critical, and this information is processed to send private alerts as electronic mail to caregivers (Figure 11b). The system also generates a considerable amount of ubiquitous big data about the rated health states of the users. For instance, the average score of five was calculated over 6080 real-time rated health-check values from 8–12 June 2021, which was followed as the indicator to determine the wellbeing of users during the infection transmission control. In brief, the system shares ubiquitous data about the conditions of the participants/patients and the inhabited indoor space. Such data are considered to be significant for remote monitoring and healthcare during COVID-19 [4,7,39,40].

Applying this real-time learning and remote monitoring system with microgrids, including various technologies, sensors, and IoT devices, has enabled lightweight and fast data generation and processing through quick and efficient prediction tasks. The system predicts each incoming and unknown 1-by-6 real-time input around 0.0048 s (Table 10). It thus provides big data about the prediction outcomes on the IoT cloud for remote monitoring and e-health services.

## 5. Discussion and Future Directions

In the selection of participants and the experiment environment, the active participation of users at the private scale is found to be significant to multiply similar care contexts with the deployed sensors and technologies for crowdsensing the IoT data from indoor spaces. It is observed, in the experiments, that distance-based motion sensors provide data for quarantine conditions by crowdsourcing user interaction and social distancing among users. Thus, the usages of motion sensors besides gas sensors, temperature, and humidity sensors are regarded with primary importance in this research in correlating the critical data about the user activity and the condition of indoor space for healthcare. Additionally, Bluetooth devices have enabled the usage of microgrids to be controlled by other mobile and electronic devices that can be recognized in the near territory. The deployed Bluetooth device enables control of the microgrid by mobile phone applications that send sound and text commands to Microgrid Z while being controlled by Arduino IDE software. Accordingly, Microgrid Z is designated as a distinct device for infection transmission control and tracing risky interactions with people using paired devices with Bluetooth, using healthcare applications [9], and with similar applications to synchronize and recognize similar microcontrollers. Moreover, Microgrid Z provides a separate LAN-based monitoring system that saves data about the measured basic metabolic values, thus enabling the recorded values to be shared in e-health applications that can also be used in hospitals (Figure 11c).

The multiplicity of data from different sensors, merged and sent as unique IoT data, enables the development of the learning models via experimental analyses that result in a developed real-time learning and monitoring system. In that regard, the physical installation of microgrids in the experiment environment influences the outcomes of experiments and the development of learning models in depth. The fixed angle between the microgrids, *theta* *(θ)*, and the angle of the agent according to the microgrids, *phi (Φ)*, are used to derive the exact algorithms in identifying some common actions and validating the incoming inputs as correlated with regard to each other. Regression models are applied further in the validation of motion tracking data and predicting the correlating changes between environmental data from the indoor space. For instance, the outcomes of regression learning tasks indicate that GPR learning models produce better RMSE results and are the most suitable for estimating the changes in the gas sensor values of indoor air quality with respect to changes in the temperature and humidity values (Table 3). The regression learning tasks also reveal the priority among the applied microgrids: With lower RMSE results, Microgrid Y is designated to operate as the predicted (posterior) model when the correlations between inputs from two microgrids are considered (Table 5). Configuration angle, *theta (θ)*, and the angle of agents, *phi (Φ)*, also provide ample evidence for classification learning and, especially, for ECOC models that are used in distinguishing the labeled activities, which are categorized through correlated inputs and anomalies (Table 4 and Table 6).

The classification and regression tasks, applied on 1012-by-2 and 1012-by-4 inputs, also enable the determination of the composition of the dataset with n-by-6 inputs, as they also depend on the physical configuration of the microgrids (Table 4 and Table 6). The machine learning models, which are applied for classifying specific behaviors and anomalies, also provide evidence for the deep learning models to recognize the correlating data from different sensors and calling attention to critical circumstances, such as two people in the room or close interaction of the users.

The acquired IoT data, processed through stepwise development of machine learning and deep learning models, also increase the efficiency of learning models in the system. Additionally, previous experiments on learning models provide the ground truth and evidence for error regularization algorithms. These algorithms are applied to process the exchange between new incoming IoT data, false negatives, and errors in the dataset. The inputs identified by their lower prediction scores are also swapped, later, with incoming inputs for optimizing the datasets and the neural networks in the system (Table 8 and Algorithm 1).

The versatility of the real-time system for monitoring and alarming can be emphasized by the fact that, even though the multilayered four neural networks are discretely optimized with similar accuracy results (Table 8), they perform diversely to filter and save the same real-time data (Table 9). The benefits of the multilayered-tandem configuration of the sequential neural networks are especially regarded in predicting the categorical activities that need sequence-based tracking, as in the classification labels 15, 17, and 20 in Table 9. Thus, the experimental results in developing the real-time learning system have enabled the improvement in the training and validation data to re-train the developed learning models in the system by following the responses of four neural networks for each labeled categorical activity input (Table 9). The improvements have enabled the system to maintain its accuracy throughout the experiments in real-time (Table 9). Finally, the system has sent over 110,000 pieces of real-time IoT data, which could be viewed publicly [61], and evolved by learning and predicting the activity of users at the private scale during the pandemic.

**Figure 12:**
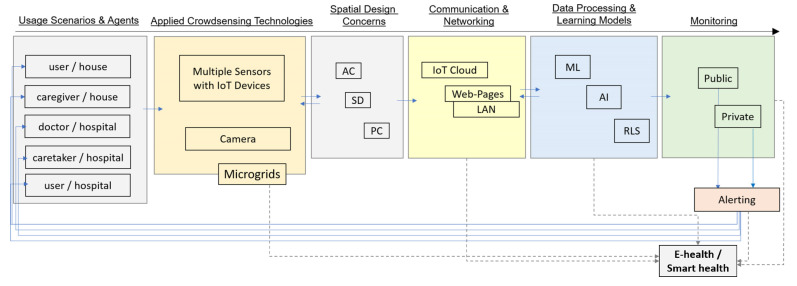
The potential scope of the research with regard to the Smart Health Services.

An equivalent number of rated health-check values was also recorded as big data, which is significant for healthcare systems to be used as an indicator in predicting the health condition and wellbeing of patients and users. The developed real-time learning and monitoring system is differentiated from other smart health applications [9,64] because it is able to record the crowdsourced data from indoor spaces along with ubiquitous health-state values rated by the participants as in the experiments from 8–12 June 2021. Hence, the real-time learning and monitoring system generates big data at the private scale that some crucial information is also registered to the e-healthcare systems and smart healthcare services about the wellbeing of users [9,12,19,48,64,65] (Figure 10a,c, Figure 11 and Figure 12). The networking capacity of this centralized learning and monitoring system is designated to merge data from multiple microgrids, so it can acquire data from different special care contexts via decentralized and autonomous IoT devices (Figure 3). It is also significant to regard microcontrollers with sensors as being energy-efficient and affordable technologies to be applied in smart spaces and infrastructure [8,13,14,15,39,54,66,67,68]. Thus, this real-time learning and monitoring system has the potential to be multiplied at decentralized IoT nodes and in the scope of smart healthcare systems, buildings, and facilities, such as hospitals, to have larger-scale centralized e-health and smart health services (Figure 12).

**Figure 13:**
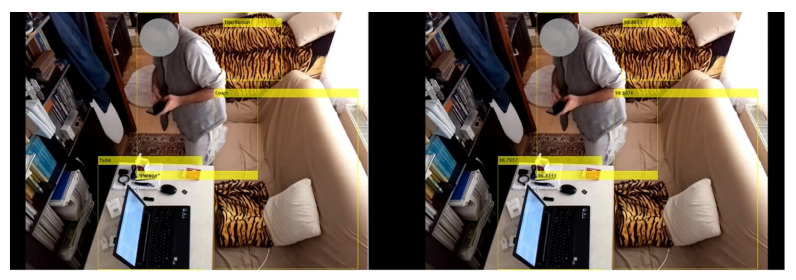
Fast R-CNN model is deployed for labeling the objects and people in the experiment room with the installation of Raspberry Pi 4 & V2 Camera. Accuracy results with true labels: Blanket: 99.87%; Coach: 98.61%; Person: 96.83%; Table: 96.77%. The elapsed time for detecting objects and people in this frame is around 9.48 s.

The limitations and future work of this study are discussed as follows: The physical configuration constraints of the experiment environment also restrict the variety of user behavior, which repeats in a similar lifecycle. Nevertheless, recurring lifecycles are recognized as persisting user activities to send private alerts, promising to develop varieties of new alerting systems. Imaging devices, wearables, Wide Area Network (WAN), and diagnostic technologies measuring glucose, blood pressure, and heart rate are also explored as other possible technologies [49,50,60] to log the basic metabolic state values in smart health systems periodically [9,19,64,65]. Some of these technologies, such as imaging devices and diagnostic technologies, are tested but not directly applied in the experiments. For instance, Raspberry Pi 4 computer with a V2 camera (Element14, Leeds, UK) is tested in real-time via an IoT connection to the learning system. In this test, the system is run with a trained fast R-CNN model, which is not actively applied in the experiments (Figure 13).

It is observed in the experiments that the frame capturing feature of image acquisition devices has the potential to be applied to healthcare services by developing a quick real-time learning system to categorize the user activity. However, imaging devices are not directly applied in these experiments to ensure privacy and efficiency, as they use more computational time and memory and cannot enable to have data about air index and gas sensor values and rated health states of the user (Figure 13). Nevertheless, this multilayered real-time system includes CNN that can still be developed or replaced with R-CNN models and can be trained and used to process larger image inputs from imaging devices to be shared with national smart healthcare applications and systems that are left as an optional feature. Therefore, the multifunctional usages of R-CNN models [21,35,69,70] remain as a future work that can be applied for versatile uses in public healthcare facilities and in infrastructural development (Figure 12). The advantages of the operating system in this research can be summarized as follows:

1. The developed real-time learning system is practical and affordable because of the low-cost microgrids with sensors and IoT devices
2. The microgrids with additional technologies (Table 1) can be multiplied at the residential scale, as well as for different types of spatial usages and special care contexts, including public buildings, hospitals, and operation rooms (Figure 12)
3. The acquired inputs from different sensors via the IoT cloud are recorded as lightweight data for monitoring and healthcare, and the learning system is lightweight, fast, and efficient in monitoring, processing, and predicting activities
4. The system can be developed for multiple nodes of larger networks and services such as smart health and e-health systems (Figure 12).

## 6. Conclusions

In this research, microgrids and IoT devices enable different types of monitoring and alerting options by generating multiple correlating inputs from various sensors, and they are operated with the developed real-time learning system. The system has enabled remote monitoring and communication by IoT-based technologies and platforms, which are extremely important for monitoring infectious interactions during the pandemic. The system has provided more than 110,000 IoT-based ubiquitous big data about user activity, with the facts from the indoor space and user-rated health-state values, which are significant for wellbeing during the infection transmission control of the SARS-CoV-2 pandemic. More significantly, it is observed, in the experiments, that the need for physical observation is minimized with the help of the remote monitoring system. Implementation of sensors and IoT-based smart technologies with learning models and the real-time updating system offers greater potential to provide a flexible infrastructure for perpetually monitoring and predicting activity and ubiquitous crowdsourcing from indoor spaces for healthcare systems and services.

It is also important to emphasize the increasing efficiency of the real-time learning models with the fact that the system maintains its accuracy of around 100% in predicting the activity patterns (Table 9) and updates the training and validation data and all learning models. With the help of the multilayered-tandem configuration of this real-time learning and monitoring system, it also becomes possible to follow each acquired data and predictions from each deep learning model about the user activity synchronously [58] (Table 9). The system utilizes image and sequential datasets, as well as learning models derived from the correlated actions. Thus, the learning system can also be developed as multi-feature fusion neural networks to be applied for different usage scenarios in the scope of healthcare and public facilities. These findings describe the potential of developing smart grids and intelligent infrastructure with the increasing technological capabilities of real-time learning and prediction models for smart healthcare services and facilities.

## Figures and Tables

**Table 1:** Microgrids with sensors and other applied technologies.

Sensors	Microgrid X	Microgrid Y	Microgrid Z	ESP32	Raspberry Pi 4
Temperature & Humidity	-	DHT 11	-	-	-
Bluetooth	-	-	HC-06	Built-in	Built-in
Camera	-	-	-	-	Camera V2
Display	-	Liquid Crystal Display (LCD)	-	-	-
Gas Sensor	MQ-2	-	-	-	-
Light	Light Emitting Diode (LED)	-	-	-	-
Motion Tracking	HC-SR04	HC-SR04	-	-	-
Remote Control	Applied	-	-	-	-
Smart Card	-	-	-	-	-
Sound	-	-	Controlled by mobile applications	-	-
Wireless Connection	ESP8266	ESP8266	ESP8266	Built-in	Built-in
Wearable Devices	-	-	-	-	-

**Table 2:** Applied rating system for crowdsourcing/diagnosing the health state of the user.

Value	Explanation
1	“I need help!”
2	“I do not feel good”
3	“It is alright!”
4	“I feel good”
5	“I feel great!”
888	Not activated

**Table 3:** Outcomes of experiments on Machine Learning models: Regression Models to predict the correlating changes in Gas Sensor values.

Regression Model		RMSE
Linear regression	Linear regression	0.88
	Interactions linear	0.88
	Robust linear	0.89
	Stepwise linear	0.88
Tree	Fine tree	0.43
	Medium tree	0.57
	Coarse	0.79
SVM	Linear SVM	0.91
	Quadratic SVM	12.1
	Cubic SVM	955.97
	Fine Gaussian SVM	0.7
	Medium Gaussian SVM	0.8
	Coarse Gaussian SVM	0.87
Ensemble	Boosted trees	2.31
	Bagged trees	0.46
GPR	Squared exponential GPR	0.63
	Matern 5/2 GPR	0.63
	**Exponential GPR**	**0.39**
	Rational quadratic GPR	0.63

**Table 4:** Outcomes of experiments on Machine Learning models: Accuracy results for different classification learning models.

Classification Model		*Two Variables of 1012-by-4 Inputs*	*1012-by-2 Inputs (without Angles)*
		Accuracy	Accuracy
Free	Fine tree	93.6	96
	Medium tree	89	91
	Coarse	78.1	74.6
Discriminant	Linear discriminant	43.9	43.2
	Quadratic discriminant	92.5	96
Naïve Bayes	Gaussian	75.5	80.4
	Kernel	78.8	81.9
SVM	Linear SVM	76.3	79.2
	Quadratic SVM	94.8	97.3
	Cubic SVM	87.7	97.5
	**Fine Gaussian SVM**	95.3	**98.6**
	Medium Gaussian SVM	88.8	95.4
	Coarse Gaussian SVM	63.8	75.8
K-nearest neighbor	Fine	94.8	98.4
	Medium	92.9	95.8
	Coarse	70.8	72.9
	Cosine	82.4	82.7
	Cubic	92.7	95.3
	**Weighted**	**95.8**	98.3
Ensemble	Boosted trees	93.4	96.2
	Bagged trees	95	97.4
	Subspace KNN	74.6	76
	RUSBoosted trees	89	91.3

**Table 5:** Regression learning outcomes on the classified movement/interaction behavior.

Regression Models		RMSE Values							
		Case 1		Case 2		Case 3		Case 4 (Anomalies)	
		X *	Y **	X *	Y **	X *	Y **	X *	Y **
Linear regression	Linear regression	0.98	0.9	0.43	0.42	1.6	1.26	41.54	31.74
	Interactions linear	0.98	0.9	0.44	0.42	1.2	0.57	38.79	30.26
	Robust linear	0.98	0.9	0.53	0.5	1.6	1.26	41.64	31.85
	Stepwise linear	0.98	0.9	0.43	0.42	1.2	0.58	38.79	30.26
Tree	Fine tree	1.45	1.11	1.27	1.38	4.76	4.07	20.79	18.8
	Medium tree	2.3	2.18	2.06	2.08	5.41	6.26	29.55	22.1
	Coarse	4.96	4.67	3.97	3.77	6.21	7.93	32.72	26.27
SVM	Linear SVM	2.22	2.05	0.9	0.95	1.76	1.31	46.9	33.4
	Quadratic SVM	1.83	1.88	1.08	1.17	1.82	1.21	112.51	28.95
	Cubic SVM	5.65	1.86	1.28	1.21	2.3	2.05	1665.6	131.81
	Fine Gaussian SVM	2.23	2.57	2.96	2.6	5.83	4.81	32.49	27.98
	Medium Gaussian SVM	1.98	1.75	1.39	1.3	2.82	2.34	40.77	27.98
	Coarse Gaussian SVM	2.12	1.73	1.03	0.97	2.42	1.36	40.85	32.5
Ensemble	Boosted trees	5.01	4.58	4.54	4.37	6.01	4.59	22.01	20.88
	Bagged trees	15.79	1.17	2.99	8.81	6.44	4.8	36.81	28.77
GPR	**Squared exponential GPR**	0.96	0.89	0.21	0.19	**0.47**	**0.33**	18.05	19.37
	**Matérn 5/2 GPR**	0.97	0.88	**0.21**	**0.19**	0.48	0.33	17.69	18.04
	**Exponential GPR**	1	**0.81**	0.37	0.34	1.3	0.84	**16.74**	16.05
	**Rational Quadratic GPR**	**0.94**	0.89	0.21	0.19	0.47	0.33	16.81	**15.96**

* Response from Microgrid X. ** Response from Microgrid Y.

**Table 6:** ECOC classification results with different learner types, hyperparameters, and ranges.

ECOC Models Learner Type, Hyperparameter, Range	Validation Accuracy		Cross-Validation and Prediction Accuracy			
	1012-by-2 Inputs	1012-by-4 Inputs	1012-by-2 Inputs		1012-by-4 Inputs	
			Cross-Validation	Test	Cross-Validation	Test
Surrogate tree & gentle boost ensemble *	83.1	99.1	83.7	76.14	99.1	95.1
SVM, Kernel, Gaussian	86	98.4	81.8	75.49	98.9	92.48
KNN, Distance, Cosine	96	**99.8**	**98.5**	**94.12**	**99.9**	**96.08**
KNN, Distance, Euclidean	98.1	98.3	97.6	92.81	99.8	**96.08**
KNN, Distance weighted, Equal	**98.3**	99.7	97.6	92.81	99.8	**96.08**
KNN, Distance weighted, Inverse	**98.3**	99.7	97.6	92.81	99.8	**96.08**
KNN, Distance weighted, Squared Inverse	**98.3**	99.7	97.6	92.81	99.8	**96.08**
KNN, Standardize	98.2	98.5	97.3	92.48	**100**	93.13

* Surrogate tree is used as learner templates fitted for the gentle boost ensemble classification method. The number of binaries is set to 50 from the ensemble learners objects with 100 learners, and coding design is set to oneVSAll; the model is tested by cross-validating the ECOC classifier using 10-fold cross-validation.

**Table 7:** Examples from the initially recorded dataset with classified user activities.

	n-by-6 Inputs						Classification Labels	Recorded & Classified Activity
n-by-2 Inputs (Motion Tracking Data) & CASES			Temp. (°C)	Humidity (%)	Gas Sensor Value	User-Rated Health State		
CASE	D1 (cm)	D2 (cm)						
3	66.27	84	25.9	50	566	888	11	Caretaker occupies
1	87.3	95	26.1	49	539	888		
2	76.59	78	25.4	36	481	888	12	Ventilation
2	77.5	79	25.4	35	472	888		
2	77.02	81	25.2	33	472	888	13	Cold-dry indoor air
2	76.26	81	25.3	33	467	888		
3	78.31	88	26.1	49	533	888	14	Hot-humid indoor air
3	78.63	87	26.1	49	536	888		
3	88.8	124	26	50	542	5	15	Empty/(Going) Out
3	86.25	131	26	50	546	5		
3	98.43	134	26	48	541	888	16	(Entering) In
1	107.19	123	26	48	540	888		
4/Anomaly	75.71	67	25.7	49	501	5	17	Moving towards the bed
4/Anomaly	78.26	69	25.8	49	424	4		
3	75.61	102	25.8	50	556	2	18	Gas value & health state correlation
3	77.61	92	26	50	508	5		
4/Anomaly	66.4	101	25.9	49	585	888	19	Two people occupy the room
4/Anomaly	66.4	103	25.9	49	578	888		
4/Anomaly	77.83	68	25.9	49	425	4	20	Interaction around the patient/bed
4/Anomaly	77.11	53	25.9	49	541	5		

**Table 8:** Results of deep learning models. (a) Initial results. (b) Performance of the neural networks after error regularization and data optimization.

Data Type	Learning Model	Learn Rate	Num. of Hidden Units	Num. of Iter.	4 Cases						10 Categories		
					1012-by-2 Inputs			1012-by-4 Inputs			312-by-6 Inputs		
					Validation (Valid.) Accuracy (Acc.)	Test Acc.	Train Time (s)	Valid. Acc.	Test Acc.	Train Time (s)	Valid. Acc.	Test Acc.	Train Time (s)
(**a**)													
Image	CNN	1 × 10^−3^	256	1000	79.1	86.3	18	99.3	95.6	23	92.6	93.8	18
				30,000	98.0	92.2	472	100	97.6	632	92.6	96.9	462
Seq.	LSTM		32	200	100	97.6	14	100	100	14	100	90	21
	Bi-LSTM				100	94.1	17	100	100	17	100	80	27
	Bi-LSTM + Btc.N.				100	75	18	100	100	18	100	90	31
(**b**)													
Image	CNN	1 × 10^−3^	144	5000	100 *	100	76 *	100	100	81	**100**	**100**	**67**
Seq.	LSTM			1000	100	100	37	100	100	23	**100**	**100**	**20**
	Bi-LSTM				100	100	61	100	100	32	**100**	**100**	**25**
	Bi-LSTM + Btc.N.				-	-	-	-	-	-	**100**	**100**	**57**

*: 100% validation accuracy is achieved at 7500 iterations in 145 s with 16 hidden units.

**Table 9:** Exemplar results of the Real-Time Learning System.

Prediction Score Threshold: 0.89						Prediction Score Threshold: 0.8				
Training Time (s)	67	20	25	57	169	The system’s overall performance in five consecutive days				
Classification Labels	CNN	LSTM	Bi-LSTM	Bi-LSTM + Btc.N.	Total Update	8 June 2021	9 June 2021	10 June 2021	11 June 2021	12 June 2021
11	1	1	-	-	2	0	0	0	0	0
12	1	1	-	-	2	0	0	0	0	0
13	1	1	-	-	2	0	0	0	0	0
14	1	1	-	-	2	0	0	0	0	0
15	1	-	-	1	2	134	705	644	709	685
16	1	1	-	-	2	0	0	0	0	0
17	1	-	-	1	2	61	201	203	203	214
18	1	-	-	-	1	270	485	495	528	527
19	1	1	-	-	2	0	0	0	0	0
20	1	-	1	-	2	0	0	0	0	14
Total Update	10	6	1	2	19	465	1391	1342	1440	1440
Total Input	10	10	4	3	20	465	1392	1343	1440	1440
Accuracy (%)	100	-	-	-	95	100	99.93	99.93	100	100

**Table 10:** Prediction durations of the real-time learning system.

	Number of Inputs				
n-by-6 inputs at a time, n:	1	50	100	500	1000
average prediction duration (seconds)	0.0048	0.1848	0.2874	1.1568	2.5390
duration per 1-by-6 input (seconds)	0.0048	0.0037	0.0029	0.0023	0.0025

## Abbreviations

Acc.	Accuracy
AI	Artificial Intelligence
Btc.N.	Batch Normalization
Bi-LSTM	Binary Layered Long-Short Term Memory
CNN	Convolutional Neural Networks
ECOC	Error-Correcting Output Codes
ELM	Extreme Learning Machine
GPR	Gaussian Process Regression
GPS	Global Positioning System
HTML	Hyper-Text Markup Language
IoT	Internet of Things
Iter.	Iteration
KNN	K-means Clustering for the Nearest Neighbor
LAN	Local Area Network
LCD	Liquid Crystal Display
LED	Light Emitting Diode
LSTM	Long-short Term Memory
ML	Machine Learning
Num.	Number
PCA	Principal Component Analysis
RLS	Real-Time Learning System
RMSE	Root-Mean-Squared-Error
Seq.	Sequence
SSID	Service Set Identifier
STA	Stationary Point
SVM	Support Vector Machine
Temp.	Temperature
Valid.	Validation
WAN	Wide Area Network
